# Prognostic value of biomarkers EpCAM and αB-crystallin associated with lymphatic metastasis in breast cancer by iTRAQ analysis

**DOI:** 10.1186/s12885-019-6016-3

**Published:** 2019-08-23

**Authors:** Liang Zeng, Xiyun Deng, Jingmin Zhong, Li Yuan, Xiaojun Tao, Sai Zhang, Yong Zeng, Guangchun He, Pingping Tan, Yongguang Tao

**Affiliations:** 1 Department of Pathology, Guangzhou Women and Children’s Medical Center, Guangzhou Medical University, Guangzhou, Guangdong China; 2 Key Laboratory of Translational Cancer Stem Cell Research, Hunan Normal University, Changsha, Hunan China; 3 Department of Pathology, Union Hospital, Tongji Medical College, HuaZhong University of Science and Technology, WuHan, China; 4 Department of Pharmacy, Hunan Normal University School of Medicine, Changsha, Hunan China; 5 Department of Oncology, Institute of Medical Sciences, Xiangya Hospital, Central South University, Changsha, Hunan China; 6 College of Life Science, Hunan Normal University, Changsha, Hunan China; 7 Department of Pathology, Hunan Cancer Hospital & The Affiliated Cancer Hospital of Xiangya School of Medicine, Central South University, Changsha, Hunan China; 8 Key Laboratory of Carcinogenesis and Cancer Invasion, Ministry of Education, Key Laboratory of Carcinogenesis, Ministry of Health, Cancer Research Institute, Xiangya Hospital, Central South University, Changsha, Hunan China

**Keywords:** Breast cancer, Metastasis, EpCAM, FADD, NDRG1, αB-crystallin, Biomarker, iTRAQ proteomic analysis

## Abstract

**Background:**

Metastasis is responsible for the majority of deaths in a variety of cancer types, including breast cancer. Although several factors or biomarkers have been identified to predict the outcome of patients with breast cancer, few studies have been conducted to identify metastasis-associated biomarkers.

**Methods:**

Quantitative iTRAQ proteomics analysis was used to detect differentially expressed proteins between lymph node metastases and their paired primary tumor tissues from 23 patients with metastatic breast cancer. Immunohistochemistry was performed to validate the expression of two upregulated (EpCAM, FADD) and two downregulated (NDRG1, αB-crystallin) proteins in 190 paraffin-embedded tissue samples. These four proteins were further analyzed for their correlation with clinicopathological features in 190 breast cancer patients.

**Results:**

We identified 637 differentially regulated proteins (397 upregulated and 240 downregulated) in lymph node metastases compared with their paired primary tumor tissues. Data are available via ProteomeXchange with identifier PXD013931. Furthermore, bioinformatics analysis using GEO profiling confirmed the difference in the expression of EpCAM between metastases and primary tumors tissues. Two upregulated (EpCAM, FADD) and two downregulated (NDRG1, αB-crystallin) proteins were associated with the progression of breast cancer. Obviously, EpCAM plays a role in the metastasis of breast cancer cells to the lymph node. We further identified αB-crystallin as an independent biomarker to predict lymph node metastasis and the outcome of breast cancer patients.

**Conclusion:**

We have identified that EpCAM plays a role in the metastasis of breast cancer cells to the lymph node. αB-crystallin, a stress-related protein that has recently been shown to be important for cell invasion and survival, was identified as a potential prognostic biomarker to predict the outcome of breast cancer patients.

**Electronic supplementary material:**

The online version of this article (10.1186/s12885-019-6016-3) contains supplementary material, which is available to authorized users.

## Background

Breast cancer is the most frequently diagnosed cancer and the leading cause of cancer death among females worldwide [1]. While the incidence rates are generally higher in more developed areas, such as North America and Australia, the incidence of breast cancer in developing countries has been increasing in recent years. In China, breast cancer has become the most common cancer in females and the leading cause of cancer-related death in younger women, especially in highly urbanized regions, which is possibly due to changes in lifestyle and reproductive behavior [2, 3]. With breast cancer, it is not the primary tumors but the metastasis that is responsible for the death of over 90% of breast cancer patients [4, 5]. Some breast cancer patients who initially present with distant metastases and resection are diagnosed with late-stage disease that is nearly incurable. It is possible that the seeds of metastasis are sown at a very early stage in the primary tumor development in the breast [5–8]. Other patients, who have no detectable metastases at the time of diagnosis, ultimately develop metastatic lesions, often months or years after the initial diagnosis [9, 10]. Therefore, the identification of metastasis-related factors warrants further investigation.

Enormous efforts have been made in identifying metastasis-related factors that can be used as prognostic markers to predict the transition from primary to systemic diseases [11–15]. Established prognostic factors that have been confirmed to be involved in breast cancer metastasis include tumor size, axillary lymph node status, and histological grade/subtype. New potential prognostic biomarkers of breast cancer metastasis are continuously being uncovered, which include uPA/PAI1, ER, PR, HER2/ErbB2, circulating tumor cells, the presence of epithelial cells in the bone marrow [12, 16], E-cadherin [17] and, more recently, nucleobindin-2 [18]. Unfortunately, each of these prognostic markers has limited prognostic value in only certain subgroups of patients with breast cancer. Moreover, metastasis to the lymph node, primarily the axillary nodes, is the earliest sign of the metastatic spread of breast cancer [19] and this process occurs at a higher rate than any single distant organ metastasis [20]. In addition to the well-known CXCL12/CXCR4 axis in directing the migration of breast cancer cells through the lymphatics [21, 22], very few studies have been conducted to identify biomarkers associated with the lymph metastasis of breast cancer.

Profiling the tumor tissue proteomics provides important information of biomarker discovery. This potentially useful strategy, however, is limited by the sensitivity of the currently available methods [16]. Isobaric tags for relative and absolute quantitation (iTRAQ) has been widely employed in quantitative proteomic studies in complex biological systems [23, 24] and has been successful in the characterization of protein bioindicators of diverse effects [25]. Recently, the combination of iTRAQ isobaric labeling, multidimensional liquid chromatography and ultrahigh resolution mass spectrometry has been used to identify tumor biomarkers in cancer, including breast cancer [26–30]. In this study, primary breast tumor tissues and paired lymph node metastases from breast cancer patients were analyzed in parallel by the quantitative iTRAQ proteomic method. Four differentially regulated proteins were validated by immunohistochemistry. Through further clinicopathological correlation and bioinformatic studies, we identified αB-crystallin as a potential prognostic biomarker to predict the occurrence of lymph metastasis and the clinical outcome of breast cancer patients.

## Methods

### Human subjects

This study was approved by the Research Ethics Committee of Central South University, China, and informed consent was obtained from all of the patients. All patients were diagnosed by two senior pathologists as invasive breast cancer (invasive ductal carcinoma or invasive lobular carcinoma) without radiotherapy or chemotherapy before surgery.

#### Mass collection methods for breast cancer

Select the cases with large lesions (> 1.5 cm × 1.5 cm × 1 cm) which were diagnosed as breast cancer by frozen section. Tissue samples were cut the tumors (> 0.5 cm × 0.5 cm × 0.5 cm) and preserved them in liquid nitrogen. We then decided whether to join the group according to routine diagnosis and lymph node metastasis.

#### Methods for collecting lymph node metastases

The lymph nodes with the largest diameter (> 1 cm) were selected, the adipose tissue around the lymph nodes was removed, the lymph nodes were cut along the largest diameter, and the color of the section was observed by naked eyes. The selected lymph nodes were divided into two parts, half of which were stored in liquid nitrogen, and the other half were stained with H&E and observed under a microscope to determine whether the lymph nodes really existed. In breast cancer metastasis, the criterion for admission was that metastatic cancer accounted for more than 90% of lymph nodes. The collected breast cancer tissues and matched metastatic lymph nodes were preserved in liquid nitrogen.

### iTRAQ proteomics

Twenty-three paired fresh primary tumors and metastatic axillary LNs were collected from Hunan Cancer Hospital between November 2013 and March 2014. Each collected tissue sample was divided into two parts; one part was used for routine pathological examination, and the other part was stored in liquid nitrogen for the proteomic study. To minimize the influence of residual lymphoid tissues on protein identification, only the axillary LNs with > 95% neoplastic cells according to H&E examination were used for the proteomic study. Relative quantitative proteomics was performed using the Fitgene iTRAQ Proteomics Platform (http://www.fitgene.com) according to the standard procedure [28, 30]. Briefly, the prepared lysates (200 μg) were treated with 4 μL of reducing reagent for 1 h at 60 °C and then blocked with 2 μL of cysteine blocking reagent for 10 min at room temperature. After centrifugation, the supernatant was collected and incubated with trypsin and TEAB overnight at 37 °C. The samples were then mixed with the iTRAQ reagents and subjected to two-dimensional LC-MS/MS analysis and a database search. An expression change greater than 1.5-fold was considered a difference between the primary tumor tissues and the paired metastatic LN tissues.

The raw data acquired from LC-MS/MS was processed with AB Sciex ProteinPilot 4.0 (AB Sciex, Concord, Ontario, Canada), and protein identification and quantification were achieved by searching the UniProt database (Release 2014.5.14). Proteomics profiling and database searching based on the TripleTOF® 5600+ System (AB Sciex) and ProteinPilot 4.0 (AB Sciex) were performed following the manufacturer’s recommendations. The parameters were set as follows: Unused ≥1.3; Credibility ≥95%; C.V. ≤ 0.5; AVG. ≥ 1.5 or ≤ 0.67; T.TEST < 0.05; Peptides (95%) ≥ 4. To ensure the reliability and stability of the reported data, we performed the following steps for data quality control. First, before database searching, we selected “Run False Discovery Rate Analysis” in the software AB Sciex ProteinPilot for FDR control. Second, we removed the results identified by the reverse database. Third, we removed those proteins with extremely high or low ratios. Finally, we removed those proteins with abnormal quantification between technical repetition and biological repetition.

The coefficients of variation (CV) of biological repetition were analyzed for data from different groups of samples. By observing the experimental data, when the coefficient of variation is within (+ 50%), 60% of the identified proteins can be covered. Most of the data exceeding the coefficient of variation are caused by individual differences of organisms. In subsequent analysis, this part of data will be excluded from the scope of analysis. The mass spectrometry proteomics data have been deposited to the ProteomeXchange Consortium via the PRIDE [31] partner repository with the dataset identifier PXD013931.

### Immunohistochemical analysis

A total of 106 paired paraffin-embedded tissue samples with lymph node metastasis were obtained from female patients with breast disease who were operated on in Hunan Cancer Hospital between May 1996 and May 2008. None of the patients underwent preoperative chemotherapy or radiotherapy. The tissue samples were fixed with 10% formaldehyde in PBS, embedded in paraffin and cut into consecutive 4-μm sections. Breast cancer was staged according to the Nottingham modified program of Bloom-Richardson scoring system.

For immunohistochemistry, a two-step polymer-based detection method (EnVison™) was used according to our recently published protocol [18]. The primary antibodies (all diluted 1:200) were rabbit monoclonal antibodies obtained from Abcam (Cambridge, MA, USA) (EpCAM [ab124825], FADD [ab108601], αB-crystallin [ab76467]) or CST (Danvers, MA, USA) (NDRG1 [#9485]). The staining was examined by two senior pathologists, and the total immunostaining score (TIS) was calculated as described.

### Clinicopathological correlation study

A total of 190 breast cancer patients admitted to Hunan Cancer Hospital between May 1996 and March 2005 were followed up for over 10 years, and the clinicopathological parameters, including age at diagnosis, tumor size, axillary node status, clinical stage, histological type/grade, ER/PR/HER2 status, and menstruation history, were recorded. These parameters were correlated with the expression levels of the four metastasis-associated proteins.

### GEO analysis

The difference in the expression levels of αB-crystallin between normal breast tissues and breast cancers was analyzed online in the Gene Expression Omnibus (GEO) profile (https://www.ncbi.nlm.nih.gov/geo/) using the search terms of “invasive breast cancer” and “*CRYAB*”.

### Statistical analysis

The statistical analysis was performed using SPSS 2.0 Software. A Wilcoxon signed-rank test was used to compare the expression of the metastasis-associated proteins between the paired primary tumors and the metastatic lesions of breast cancer on immunohistochemistry. A chi-square (χ^2^) test was used to evaluate the metastasis-associated proteins with the clinicopathological parameters. Survival analysis was performed using the Kaplan-Meier method. The Student’s *t* test was used to compare the mRNA expression of FADD and αB-crystallin between normal breast and breast cancer tissues from the GEO profile. A *p* value of less than 0.05 was considered statistically significant.

## Results

### Identification of lymph metastasis-associated proteins in breast cancer patients

To identify the proteins associated with lymph metastasis of breast cancer, we first analyzed 23 paired primary tumors and axillary lymph node metastases from patients with metastatic breast cancer using iTRAQ-based proteomic analysis. The quantitative data are presented in Additional file 3: Table S1. A total of 637 differentially regulated proteins (397 upregulated and 240 downregulated) between the primary sites and the lymph node metastases of breast cancer were identified based on a 95% confidence interval and a difference ratio of ≥1.5 for up-regulated protein, and ratio ≤ 0.67 for down-regulated. The top 30 upregulated and downregulated proteins are presented in Additional file 4: Table S2 and Table S3, respectively.

To gain insights into the biological and molecular characteristics of these proteins, gene ontology (GO) analysis was performed on the differentially regulated proteins. An analysis of the biological process annotations of the 397 proteins that were upregulated in metastatic sites is shown in Additional file 1: Figure S1A. These proteins were predominantly involved in cellular nitrogen compound metabolism and biosynthesis, followed by signal transduction, small molecule metabolism, and stress responses. The GO enrichment analysis of cellular components indicated that these upregulated proteins were primarily distributed in the nucleus and the cytoplasm (Additional file 1: Figure S1B). In terms of molecular functions, the majority of these upregulated proteins were involved in binding activities, such as RNA binding and ion binding (Additional file 1: Figure S1C). The 240 proteins that were downregulated in lymph node metastases were primarily associated with signal transduction, anatomical structure development, stress response, and cell differentiation (Additional file 1: Figure S1D). For cellular distribution, the downregulated proteins were predominantly localized in the extracellular region, the organelles, and the cytoplasm (Additional file 1: Figure S1E). The most significant molecular function of these downregulated proteins was ion binding (Additional file 1: Figure S1F).

### Validation of differentially regulated proteins

We filtered out four proteins (two upregulated proteins and two downregulated proteins) for further validation. These proteins were chosen based on the following criteria: 1) they had a fold-change of greater than 1.5 (for the upregulated proteins) or less than 0.67 (for the downregulated proteins); 2) they had a peptide number of greater than 3 in the iTRAQ identification; and 3) they are known to be related to cancer cell invasion/metastasis based on previous studies. These four proteins were EpCAM (epithelial cell adhesion molecule) [32], FADD (Fas-associated death domain protein) [33], NDRG1 (N-myc downstream-regulated gene 1) [34] and αB-crystallin (Alpha-crystallin B chain) [35], and their ratios of metastatic vs. primary tumor sites were 1.85, 1.51, 0.33, and 0.34, respectively. The mass annotated product ion spectra of these four proteins were obtained (data not shown). The biological processes, cellular locations, and molecular functions of these four individual proteins (Additional file 4: Table S4**)** were analyzed using the UniProt knowledgebase (http://www.uniprot.org/), which was in agreement with the abovementioned GO analysis results.

**Fig. 1:**
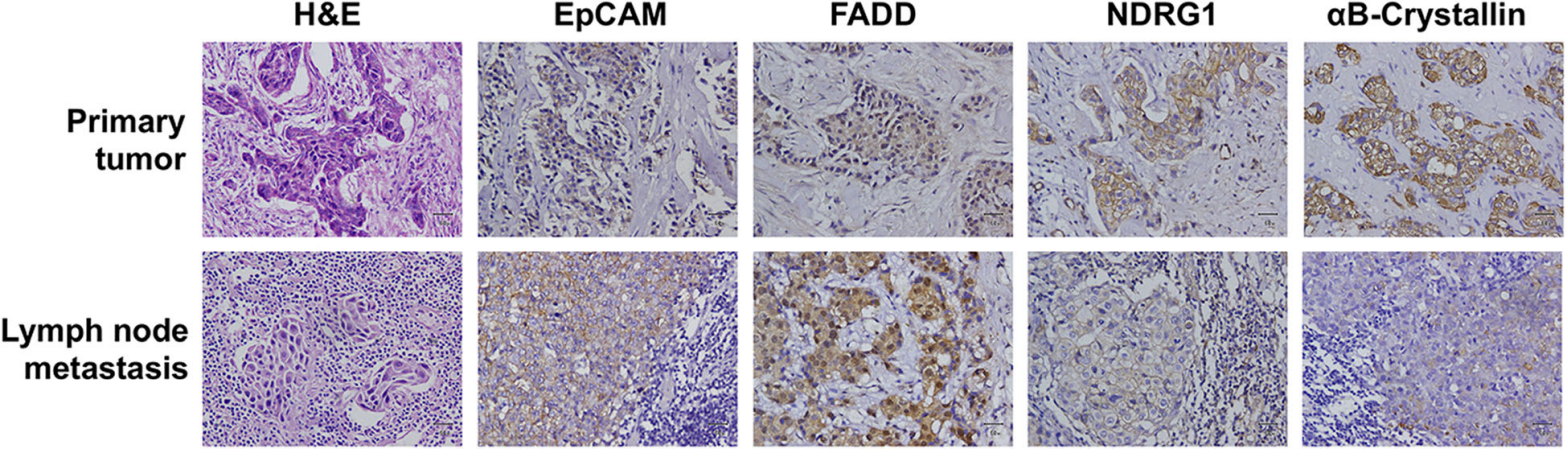
Immunohistochemical analysis of the expression of four breast cancer metastasis-associated proteins. The expression levels of EpCAM, FADD, NDRG1, and αB-crystallin were evaluated by the immunohistochemical staining of paraffin-embedded paired primary and metastatic tissue sections that were obtained from patients with metastatic breast cancer

Next, we used immunohistochemistry to verify the expression of the four breast cancer lymph metastasis-associated proteins in 106 cases of paraffin-embedded paired primary tumors and lymph metastasis tissues obtained from metastatic breast cancer patients. The representative staining images are presented in Fig. 1, and the quantitatively analyzed results, which are presented as total immunostaining score (TIS), are summarized in Table 1. As shown in Fig. 1, most of the EpCAM was localized on the plasma membrane, which is in agreement with its known cellular localization. FADD was primarily localized in the cytoplasm and the nucleus. NDRG1 was located in the plasma membrane and the cytoplasm. The αB-crystallin protein was primarily expressed on the plasma membrane and in the cytoplasm. Consistent with the iTRAQ data, NDRG1 and αB-crystallin were downregulated at the metastatic sites compared with the primary tumors in terms of TIS (Table 1) (*P* = 0.0003 [NDRG1] or *P* = 0.046 [αB-crystallin]). However, the expression levels of EpCAM and FADD were also lower at the metastatic sites compared with the primary tumors (*P* = 0.0005).

**Table 1:**
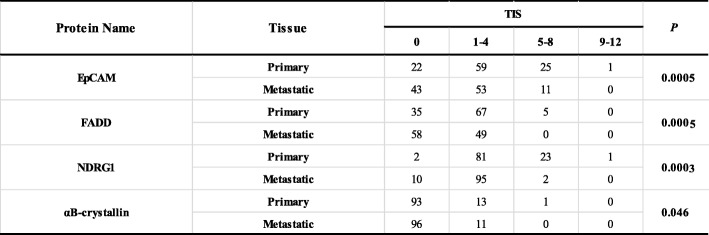
Summary of the expression of the four metastasis-associated proteins in the paired primary and metastatic tissues of breast cancer

### Correlation of metastasis-associated proteins with the clinicopathological features of breast cancer patients

To clarify the clinical relevance of the proteins identified from iTRAQ proteomics that were associated with lymph metastasis, we analyzed the relationship between these four proteins and the clinicopathological parameters of 190 cases of breast cancer patients. We showed that EpCAM was not correlated with any of the clinicopathological parameters examined (Table 2). However, FADD expression was positively correlated with a younger age at diagnosis (*P* = 0.049) and lymph node metastasis (*P* = 0.003). NDRG1 expression was correlated with worse histological grade (*P* = 0.041) but not with lymph node metastasis (*P* = 0.655). αB-crystallin expression was inversely correlated with lymph node metastasis (*P* < 0.001), clinical stage (*P* = 0.001), histological grade (*P* = 0.037), ER (*P* < 0.001), and PR status (*P* = 0.007).

**Table 2:**
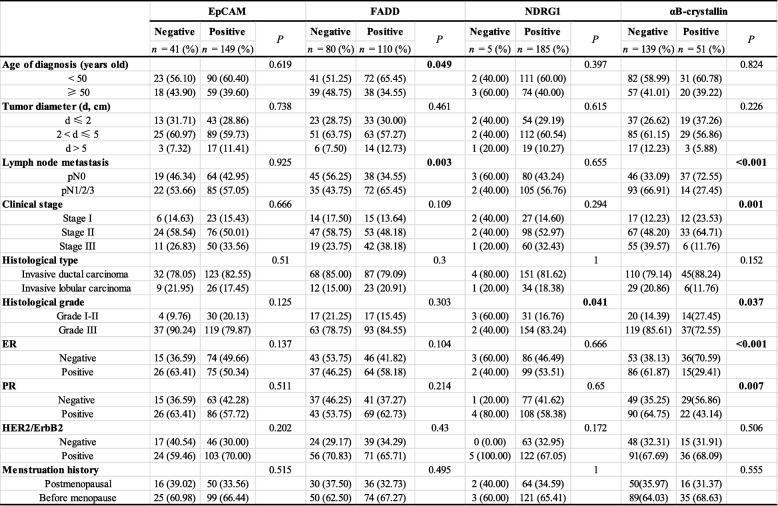
The association between the four metastasis-associated proteins and the clinicopathological features of 190 breast cancer patients

### Association of metastasis-associated proteins with overall survival of breast cancer patients

**Fig. 2:**
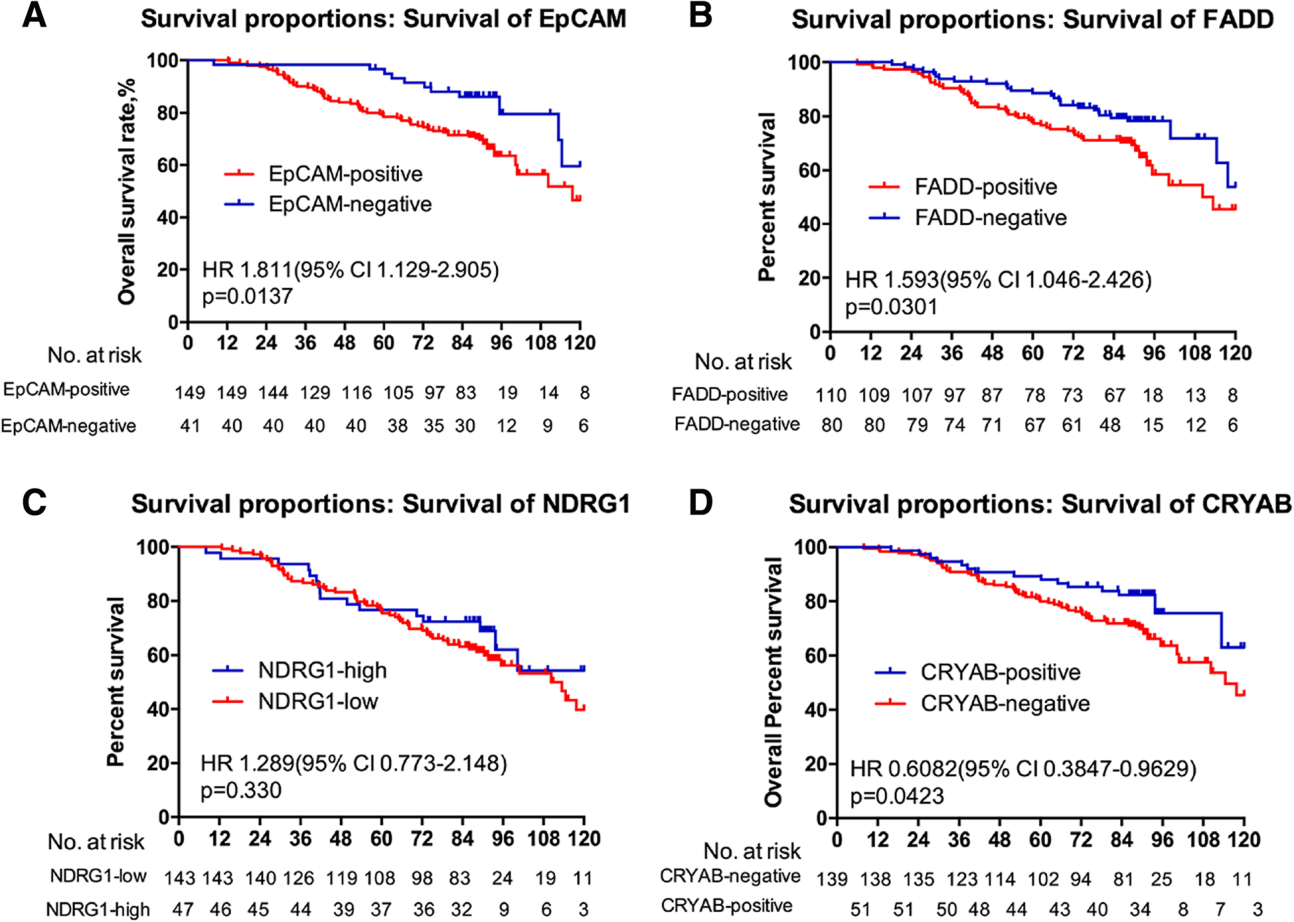
The association between four metastasis-associated proteins and the overall survival of breast cancer patients. Kaplan-Meier plots of the association between the expression of EpCAM (**a**), FADD (**b**), NDRG1 (**c**), and αB-crystallin (**d**) and the overall survival probability of breast cancer patients

**Fig. 3:**
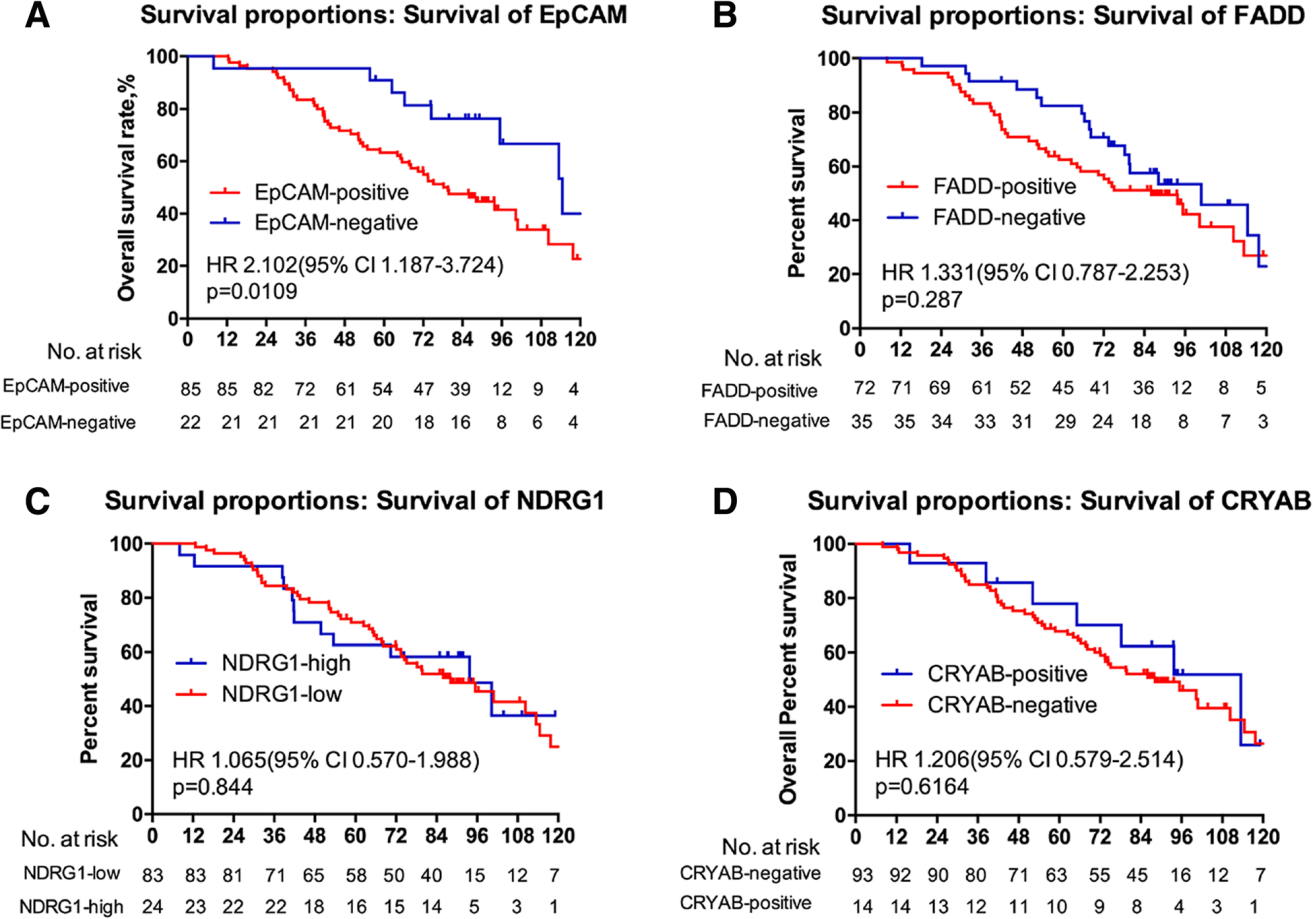
The association between four metastasis-associated proteins and the overall survival in breast cancer patients with metastasis. Kaplan-Meier plots of the association between the expression of EpCAM (**a**), FADD (**b**), NDRG1 (**c**), and αB-crystallin (**d**) and the overall survival probability in breast cancer patients with metastasis

In addition, we followed up 190 breast cancer patients for over 10 years and conducted a survival analysis for the positivity of expression (EpCAM, FADD, and αB-crystallin) or the level of expression (NDRG1) in the primary tumor sites. The results revealed that the patients who had positive expression of EpCAM or FADD survived for a shorter time compared with those with negative expression (Fig. 2a-b). Those who had positive expression of αB-crystallin survived longer than those with negative expression (Fig. 2d). However, the expression level of NDRG1 had no prognostic value for breast cancer patients (Fig. 2c). Moreover, the prognostic value of EpCAM only applied to patients with lymph node metastasis (Fig. 3a-d). Univariable analysis linked with tumor diameter, TNM stage and histology stage and type, but multivariable analysis assigned significance only to histology type (lobular carcinoma vs. duct carcinoma) (Table 3).

**Table 3:** Univariate and Multivariate Analysis by a Cox Proportional Hazards Regression Model in Cohort

Variable	OS			
	Univariate		Multivariate	
	HR (95% CI)	*P* Value	HR (95% CI)	*P* Value
Age, years (> 45 vs. ≤ 45)	0.898 (0.581–1.390)	0.630	NA	
ER (positive vs. negative)	1.114 (0.711–1.745)	0.637	NA	
PR (positive vs. negative)	1.213 (0.775–1.899)	0.398	NA	
CrebB-2 (positive vs. negative)	1.128 (0.705–1.806)	0.615	NA	
Menstrual history (presence vs. absence)	1.381 (0.851–2.241)	0.191	NA	
Operation		0.649	NA	
Modified radical mastectomy vs. radical correction	1.150 (0.727–1.820)	0.550	NA	
Other operation vs. radical correction	0.642 (0.155–2.663)	0.542	NA	
FADD (positive vs. negative)	1.580 (0.995–2.509)	0.053	NA	
NDRG1 (low vs. high)	1.302 (0.762–2.226)	0.335	NA	
CRYAB (positive vs. negative)	1.561 (0.902–2.701)	0.112	NA	
Tumor diameter, cm		0.072		NS
> 5 vs. > 2 and ≤ 5	1.923 (1.019–3.636)	0.043		
> 5 vs. ≤2	2.230 (1.093–4.549)	0.027		
TNM stage		< 0.0001		NS
III vs. I	4.329 (1.824–10.273)	0.001		
III vs. II	2.101 (1.333–3.311)	0.001		
Histology stage (poorly differentiation vs. high-middle differentiation)	2.286 (1.100–4.751)	0.027		NS
Histology type (lobular carcinoma vs. duct carcinoma)	1.720 (1.025–2.886)	0.040	1.846 (1.093–3.118)	**0.022**
Lymph node metastasis (presence vs. absence)	2.810 (1.694–4.662)	< 0.0001	2.801 (1.688–4.649)	**< 0.0001**
EpCAM (positive vs. negative)	2.306 (1.218–4.367)	0.010	2.585 (1.351–4.944)	**0.004**

Data in bold are *P* values < 0.05

### Downregulation of αB-crystallin mRNA expression in breast cancer

Finally, to examine whether αB-crystallin (gene name: *CRYAB*) was also involved in human breast cancer development, using the public database, we reviewed the mRNA expression of *CRYAB* in normal breast and invasive breast cancer tissues in Gene Expression Omnibus (GEO) (Expression Profile GDS3324). The results are presented in Additional file 2: Figure S2. The expression of *CRYAB* was significantly lower in breast cancer tissues compared with normal breast tissues (*P* = 0.001). We further found that the level of expression of αB-crystallin was indeed lower in breast cancer tissues compared with benign breast lesions, with metastatic breast cancer having the lowest expression (Table 4). These findings support the tumor-suppressive role of αB-crystallin in the development of breast cancer.

**Table 4:** Summary of the expression of CRYAB in different stages of breast tissues

Tissue	TIS				*P*
	0	1–4	5–8	9–12	
Benign	1	24	16	6	0.0003
Non-metastatic	46	28	6	3	
Metastatic	189	24	1	0	

## Discussion

Metastasis is one of the most important factors that causes the death of patients with breast cancer. Detection of breast cancer metastasis at the earliest possible stage is critical for the successful management of breast cancer progression. Therefore, it is very important to search for effective biomarkers for breast cancer metastasis and prognosis. In proteomic comparative studies of breast cancer metastasis, with tumor tissue as the research object, the commonly used method is based on the comparison of lymph node metastasis or other organ metastases, gene expression or protein expression of primary breast cancer with metastasis and without metastasis. In this study, we used the iTRAQ proteomic technique to analyze the differentially regulated proteins between the primary tumor sites and their corresponding lymph node metastases in metastatic breast cancer patients, and this comparison method can more accurately compare the differences in protein expression of breast cancer cells with varying metastatic capacity. Four proteins (EpCAM, FADD, NDRG1, and αB-crystallin) were chosen for validation by immunohistochemistry. Specially, αB-crystallin could potentially be addressed as a potential prognostic biomarker to predict the lymph node metastasis and clinical outcomes of breast cancer patients.

αB-crystallin, also called HspB5, is a member of the α-crystallin family small heat shock proteins and is an important component of the vertebrate lens [36]. In nonlens tissues, αB-crystallin is an integral part of the cellular proteostasis system, which is associated with a broad spectrum of human diseases, including cancer [37]. αB-crystallin plays an important role in stress responses, such as heat shock and radiation poisoning. As a molecular chaperone, αB-crystallin is expressed in human cells at a higher level under pathological conditions. The expression of αB-crystallin in human renal carcinogenesis, triple-negative (basal-like) breast cancer, hepatocellular carcinoma, and squamous cell carcinoma of the head and neck is related to poor prognosis [36, 37], suggesting an oncogenic role for αB-crystallin in promoting tumorigenesis. In breast cancer, αB-crystallin has been shown to be an oncoprotein that predicts poor prognosis [38–41] and resistance to neoadjuvant chemotherapy, especially for triple-negative breast cancer [40, 42]. However, the role of αB-crystallin as a tumor suppressor has also been reported [43]. These contradictory findings indicate that the role of αB-crystallin in carcinogenesis is complicated. The present study demonstrated that αB-crystallin was downregulated in the lymph metastases compared with the primary breast tumors. This finding is inconsistent with the previous finding that αB-crystallin expression promotes the brain metastasis of breast cancer [38, 44]. Recently, the majority of lymphatic and distant metastases were shown to originate differently in human colorectal cancer [45]. This phenomenon is also true for breast cancer metastasis, in which approximately 1/3 of breast cancer patients without lymph metastasis develop distant metastasis [46]. These observations suggest that the two routes of cancer spreading may occur independently and may use different sets of molecular routers to drive the metastatic spread of cancer cells through either the lymphatics or the blood vessels. Reconciling our data with the previous reports, it is possible that αB-crystallin plays a role of router to switch between lymphatic and hematogenous spreading. That is, the role of αB-crystallin in breast cancer progression needs to be reevaluated. It is speculated that αB-crystallin may function as a tumor promoter in hematogenous metastasis – to the brain, for example, but αB-crystallin may function as a tumor suppressor in lymph node metastasis. However, this speculation should be validated experimentally through in vitro and in vivo studies. Clearly, our findings further support a tumor-suppressor role for αB-crystallin in breast cancer development.

Many studies have shown that there is close link between FADD and many cancers, such as nonsmall cell lung cancer [47], gastric cancer [48] and hepatocellular carcinoma (HCC) [49]. In the first two of these cancers, the expression of FADD was correlated with lymph node metastasis and the poor prognosis of patients, and the loss of FADD expression plays an important role in HCC carcinogenesis. FADD expression is associated with T stage and perineural invasion [50]. An increase in FADD expression was shown to be associated with a higher incidence of lymph node metastasis at presentation and with a shorter DMFI when lymph node metastases are present [33]. These studies only involved the comparison between cancer and the surrounding normal tissues, whereas we focused on the differences in FADD expression between primary tumors and metastases. Using proteomic results, we determined that the expression of FADD was upregulated in metastasis. Furthermore, the IHC results revealed that there were significant differences in FADD expression between the primary tumors and metastases, but the rate of FADD-positive tumors decreased, which is inconsistent with the proteomic results. The possible reason for this inconsistency is that proteomics analyzes the relative quantity of protein expression, whereas immunohistochemistry analyzes the positive rate of protein expression, and thus results from these two methods are not always consistent. In addition, we also investigated potential correlations between FADD expression and the clinical pathological characteristics of 190 patients with breast cancer. We performed a 120-months survival analysis and found that FADD expression was associated with lymph node metastasis. Furthermore, higher expression levels of FADD were identified in patients with breast cancer, which were also correlated with a shorter survival time. These finding suggest that there is a close relationship between FADD expression and the lymph node metastasis and poor prognosis of breast cancer. Moreover, the regulatory mechanism of FADD in breast cancer metastasis warrants further investigation.

NDRG1 has been reported to function as a metastasis suppressor gene, and it is downregulated in gastric cancer [34], prostate [51, 52], pancreatic cancer [53] and breast cancers [45]. However, compared with normal tissue, NDRG1 expression was shown to be upregulated in homologous hepatocellular carcinoma [54] and oral squamous cell carcinoma [55]. In this study, all of the proteomics and IHC results revealed that NDRG1 expression was downregulated in metastases compared to the primary tumors. The expression of NDRG1 in various tissues may be affected by many factors, such as metal ions, oxygen, proto-oncogenes, tumor suppressor genes, hormones or vitamins. For example, NDRG1 expression in prostate cancer cells was shown to be affected by androgens, whereas NDRG1 expression in breast cancer cells is mainly associated with estradiol. Thus, the expression of NDRG1 is variable. In the clinical pathology and survival analysis, significant differences in NDRG1 expression were not detected in this study.

EpCAM is a transmembrane glycoprotein and appears to play a role in tumorigenesis and metastasis of carcinomas [56]. EpCAM is frequently upregulated in carcinomas but is not expressed in cancers of non-epithelial origin. At present, the FDA approves the automated cell detection method for EpCAM as biomarker, and this method has been used to detect circulating tumor cells in patients with breast [57], prostate [32, 58] and esophageal cancer [59]. The expression of EpCAM was shown to be high in laryngeal carcinoma but low in bone marrow as a metastatic niche for disseminated cancer cells [60]. These findings are consistent with our IHC results. However, EpCAM expression was increased in the metastatic group compared to the nonmetastatic group according to both iTRAQ and the proteomics analysis. Furthermore, the survival analysis showed that the survival rate was lower in the EpCAM-positive group. Therefore, the expression of EpCAM should be further clarified in breast cancer metastasis. Taken together, these data suggest that EpCAM plays a critical role in the metastatic process of breast cancer.

## Conclusions

In summary, we discovered differentially regulated proteins between the primary breast tumors and their lymph node metastatic sites using the iTRAQ proteomics analysis. Through further immunohistochemical study, clinicopathological correlation analysis, and GEO profiling, we identified αB-crystallin as an independent biomarker to predict the outcome of breast cancer patients in the lymph node. Obviously, αB-crystallin plays a role in the metastasis of breast cancer cells to the lymph node, but its exact role in each step of breast cancer metastasis and the underlying signaling mechanism remain to be fully clarified. EpCAM, FADD and NDRG1 expression were shown to be associated with the progression of breast cancer, but the questions of how certain oncogenes may initiate dissemination before triggering aggressive proliferation and how tumor-suppressor pathways suppress metastasis in breast cancer warrant further investigation.

## Additional files

Additional file 1: **Figure S1.** GO analysis of the differentially regulated proteins in lymph node metastases vs. primary breast tumor tissues. The upregulated (A-C) and downregulated (D-F) proteins identified by the iTRAQ proteomics were analyzed by the GO Consortium and categorized according to their biological processes, cellular locations, and molecular functions. (TIF 5559 kb)

Additional file 2: **Figure S2.** GEO analysis of *CRYAB* mRNA expression in normal breast and breast cancer tissues. (A) The mRNA expression of *CRYAB* in normal breast tissues (*n* =5) and breast cancer tissues (*n* = 28) was analyzed from the Affymetrix Human Genome Microarray at the GEO website (https://www.ncbi.nlm.nih.gov/geoprofiles/54408377 for αB-crystallin). (B) Quantification of the mRNA expression of *CRYAB* in normal breast tissues and breast cancer tissues. (TIF 4929 kb)

Additional file 3: **Table S1.** Identification of differentially expressed proteins between primary breast cancer tissues and metastatic lymph node tissues by the iTRAQ technique. (XLS 1215 kb)

Additional file 4: **Table** **S2.** Partial up-regulated proteins in metastatic lymph node compared with primary tumor in breast cancer. **Table S3.** Partial down-regulated proteins in metastatic lymph node compared with primary tumor in breast cancer. **Table S4.** UniProt analysis of the biological processes, cellular locations, and molecular functions of the four metastasis-associated proteins. (DOCX 29 kb)

## Data Availability

The mass spectrometry proteomics data have been deposited to the ProteomeXchange Consortium via the PRIDE [1] partner repository with the dataset identifier PXD013931.

## Abbreviations

EpCAM: Epithelial cell adhesion molecule
FADD: Fas-associated death domain
GEO: Gene expression omnibus
GO: Gene ontology
HCC: Hepatocellular carcinoma
iTRAQ: Isobaric tags for relative and absolute quantitation
NDRG1: N-myc downstream-regulated gene 1
TIS: Total immunostaining score

## References

[1] Torre LA, Bray F, Siegel RL, Ferlay J, Lortet-Tieulent J, Jemal A (2015). Global cancer statistics, 2012. CA Cancer J Clin.

[2] Chen W, Zheng R, Baade PD, Zhang S, Zeng H, Bray F, Jemal A, Yu XQ, He J (2016). Cancer statistics in China, 2015. CA Cancer J Clin.

[3] Chen W, Zheng R, Zuo T, Zeng H, Zhang S, He J (2016). National cancer incidence and mortality in China, 2012. Chin J Cancer Res.

[4] Jin X, Mu P (2015). Targeting breast Cancer metastasis. Breast Cancer (Auckl).

[5] Schwartz RS, Erban JK (2017). Timing of metastasis in breast Cancer. N Engl J Med.

[6] DeMichele A, Yee D, Esserman L (2017). Mechanisms of resistance to neoadjuvant chemotherapy in breast Cancer. N Engl J Med.

[7] Harper KL, Sosa MS, Entenberg D, Hosseini H, Cheung JF, Nobre R, Avivar-Valderas A, Nagi C, Girnius N, Davis RJ (2016). Mechanism of early dissemination and metastasis in Her2(+) mammary cancer. Nature.

[8] Hosseini H, Obradovic MM, Hoffmann M, Harper KL, Sosa MS, Werner-Klein M, Nanduri LK, Werno C, Ehrl C, Maneck M (2016). Early dissemination seeds metastasis in breast cancer. Nature.

[9] O'Shaughnessy J (2005). Extending survival with chemotherapy in metastatic breast cancer. Oncologist.

[10] Spolverato G, Vitale A, Bagante F, Connolly R, Pawlik TM (2017). Liver resection for breast Cancer liver metastases: a cost-utility analysis. Ann Surg.

[11] Ohsfeldt RL, Ward MM, Schneider JE, Jaana M, Miller TR, Lei Y, Wakefield DS (2005). Implementation of hospital computerized physician order entry systems in a rural state: feasibility and financial impact. J Am Med Inform Assoc.

[12] van de Vijver MJ, He YD, van't Veer LJ, Dai H, Hart AA, Voskuil DW, Schreiber GJ, Peterse JL, Roberts C, Marton MJ (2002). A gene-expression signature as a predictor of survival in breast cancer. N Engl J Med.

[13] Chandran UR, Ma C, Dhir R, Bisceglia M, Lyons-Weiler M, Liang W, Michalopoulos G, Becich M, Monzon FA (2007). Gene expression profiles of prostate cancer reveal involvement of multiple molecular pathways in the metastatic process. BMC Cancer.

[14] Appierto V, Di Cosimo S, Reduzzi C, Pala V, Cappelletti V, Daidone MG (2017). How to study and overcome tumor heterogeneity with circulating biomarkers: the breast cancer case. Semin Cancer Biol.

[15] Ramaswamy S, Ross KN, Lander ES, Golub TR (2003). A molecular signature of metastasis in primary solid tumors. Nat Genet.

[16] Lorusso G, Ruegg C (2012). New insights into the mechanisms of organ-specific breast cancer metastasis. Semin Cancer Biol.

[17] Fry SA, Sinclair J, Timms JF, Leathem AJ, Dwek MV (2013). A targeted glycoproteomic approach identifies cadherin-5 as a novel biomarker of metastatic breast cancer. Cancer Lett.

[18] Zeng Liang, Zhong Jingmin, He Guangchun, Li Fangjun, Li Jing, Zhou Wen, Liu Wenbin, Zhang Yun, Huang Sanqian, Liu Zhihong, Deng Xiyun (2017). Identification of Nucleobindin-2 as a Potential Biomarker for Breast Cancer Metastasis Using iTRAQ-based Quantitative Proteomic Analysis. Journal of Cancer.

[19] Castle J, Shaker H, Morris K, Tugwood JD, Kirwan CC (2014). The significance of circulating tumour cells in breast cancer: a review. Breast.

[20] Lu X, Kang Y (2007). Organotropism of breast cancer metastasis. J Mammary Gland Biol Neoplasia.

[21] Patsialou A, Wang Y, Lin J, Whitney K, Goswami S, Kenny PA, Condeelis JS (2012). Selective gene-expression profiling of migratory tumor cells in vivo predicts clinical outcome in breast cancer patients. Breast Cancer Res.

[22] van’t Veer LJ, Dai H, van de Vijver MJ, He YD, Hart AA, Mao M, Peterse HL, van der Kooy K, Marton MJ, Witteveen AT (2002). Gene expression profiling predicts clinical outcome of breast cancer. Nature.

[23] Zieske LR (2006). A perspective on the use of iTRAQ reagent technology for protein complex and profiling studies. J Exp Bot.

[24] Evans C, Noirel J, Ow SY, Salim M, Pereira-Medrano AG, Couto N, Pandhal J, Smith D, Pham TK, Karunakaran E (2012). An insight into iTRAQ: where do we stand now?. Anal Bioanal Chem.

[25] Putz SM, Boehm AM, Stiewe T, Sickmann A (2012). iTRAQ analysis of a cell culture model for malignant transformation, including comparison with 2D-PAGE and SILAC. J Proteome Res.

[26] Zeidan B, Manousopoulou A, Garay-Baquero DJ, White CH, Larkin SET, Potter KN, Roumeliotis TI, Papachristou EK, Copson E, Cutress RI (2018). Increased circulating resistin levels in early-onset breast cancer patients of normal body mass index correlate with lymph node negative involvement and longer disease free survival: a multi-center POSH cohort serum proteomics study. Breast Cancer Res.

[27] Bouchal P, Dvorakova M, Roumeliotis T, Bortlicek Z, Ihnatova I, Prochazkova I, Ho JT, Maryas J, Imrichova H, Budinska E (2015). Combined proteomics and transcriptomics identifies carboxypeptidase B1 and nuclear factor kappaB (NF-kappaB) associated proteins as putative biomarkers of metastasis in low grade breast Cancer. Mol Cell Proteomics.

[28] Bouchal P, Roumeliotis T, Hrstka R, Nenutil R, Vojtesek B, Garbis SD (2009). Biomarker discovery in low-grade breast cancer using isobaric stable isotope tags and two-dimensional liquid chromatography-tandem mass spectrometry (iTRAQ-2DLC-MS/MS) based quantitative proteomic analysis. J Proteome Res.

[29] Jesneck JL, Mukherjee S, Yurkovetsky Z, Clyde M, Marks JR, Lokshin AE, Lo JY (2009). Do serum biomarkers really measure breast cancer?. BMC Cancer.

[30] Ruppen I, Grau L, Orenes-Pinero E, Ashman K, Gil M, Algaba F, Bellmunt J, Sanchez-Carbayo M (2010). Differential protein expression profiling by iTRAQ-two-dimensional LC-MS/MS of human bladder cancer EJ138 cells transfected with the metastasis suppressor KiSS-1 gene. Mol Cell Proteomics.

[31] Perez-Riverol Y, Csordas A, Bai J, Bernal-Llinares M, Hewapathirana S, Kundu DJ, Inuganti A, Griss J, Mayer G, Eisenacher M (2019). The PRIDE database and related tools and resources in 2019: improving support for quantification data. Nucleic Acids Res.

[32] Ni J, Cozzi PJ, Duan W, Shigdar S, Graham PH, John KH, Li Y (2012). Role of the EpCAM (CD326) in prostate cancer metastasis and progression. Cancer Metastasis Rev.

[33] Pattje WJ, Melchers LJ, Slagter-Menkema L, Mastik MF, Schrijvers ML, Gibcus JH, Kluin PM, Hoegen-Chouvalova O, van der Laan BF, Roodenburg JL (2013). FADD expression is associated with regional and distant metastasis in squamous cell carcinoma of the head and neck. Histopathology.

[34] Chang X, Xu X, Ma J, Xue X, Li Z, Deng P, Zhang S, Zhi Y, Chen J, Dai D (2014). NDRG1 expression is related to the progression and prognosis of gastric cancer patients through modulating proliferation, invasion and cell cycle of gastric cancer cells. Mol Biol Rep.

[35] Liu S, Yan B, Lai W, Chen L, Xiao D, Xi S, Jiang Y, Dong X, An J, Chen X (2014). As a novel p53 direct target, bidirectional gene HspB2/alphaB-crystallin regulates the ROS level and Warburg effect. Biochim Biophys Acta.

[36] Cvekl A, McGreal R, Liu W (2015). Lens development and Crystallin gene expression. Prog Mol Biol Transl Sci.

[37] Haslbeck M, Peschek J, Buchner J, Weinkauf S (2016). Structure and function of alpha-crystallins: Traversing from in vitro to in vivo. Biochim Biophys Acta.

[38] Malin D, Strekalova E, Petrovic V, Deal AM, Al Ahmad A, Adamo B, Miller CR, Ugolkov A, Livasy C, Fritchie K (2014). alphaB-crystallin: a novel regulator of breast cancer metastasis to the brain. Clin Cancer Res.

[39] Malin D, Strekalova E, Petrovic V, Rajanala H, Sharma B, Ugolkov A, Gradishar WJ, Cryns VL (2015). ERK-regulated alphaB-crystallin induction by matrix detachment inhibits anoikis and promotes lung metastasis in vivo. Oncogene.

[40] Petrovic V, Malin D, Cryns VL (2013). alphaB-crystallin promotes oncogenic transformation and inhibits caspase activation in cells primed for apoptosis by Rb inactivation. Breast Cancer Res Treat.

[41] Wang F, Chen X, Li C, Sun Q, Chen Y, Wang Y, Peng H, Liu Z, Chen R, Liu K (2014). Pivotal role of augmented alphaB-crystallin in tumor development induced by deficient TSC1/2 complex. Oncogene.

[42] Chen Z, Ruan Q, Han S, Xi L, Jiang W, Jiang H, Ostrov DA, Cai J (2014). Discovery of structure-based small molecular inhibitor of alphaB-crystallin against basal-like/triple-negative breast cancer development in vitro and in vivo. Breast Cancer Res Treat.

[43] Huang Z, Cheng Y, Chiu PM, Cheung FM, Nicholls JM, Kwong DL, Lee AW, Zabarovsky ER, Stanbridge EJ, Lung HL (2012). Tumor suppressor alpha B-crystallin (CRYAB) associates with the cadherin/catenin adherens junction and impairs NPC progression-associated properties. Oncogene.

[44] Voduc KD, Perou CM, Harrell JC, Fan C, Kennecke H, Minn AJ, Cryns VL, Cheang MCU, Nielsen TO (2015). alphaB-crystallin expression in breast Cancer is associated with brain metastasis. NPJ Breast Cancer.

[45] Bandyopadhyay S, Pai SK, Hirota S, Hosobe S, Takano Y, Saito K, Piquemal D, Commes T, Watabe M, Gross SC (2004). Role of the putative tumor metastasis suppressor gene Drg-1 in breast cancer progression. Oncogene.

[46] Weigelt B, Peterse JL, van’t Veer LJ (2005). Breast cancer metastasis: markers and models. Nat Rev Cancer.

[47] Cimino Y, Costes A, Damotte D, Validire P, Mistou S, Cagnard N, Alifano M, Regnard JF, Chiocchia G, Sautes-Fridman C (2012). FADD protein release mirrors the development and aggressiveness of human non-small cell lung cancer. Br J Cancer.

[48] Yoo NJ, Lee SH, Jeong EG, Lee JW, Soung YH, Nam SW, Kim SH, Lee JY, Lee SH (2007). Expression of nuclear and cytoplasmic phosphorylated FADD in gastric cancers. Pathol Res Pract.

[49] Tu W, Luo M, Wang Z, Yan W, Xia Y, Deng H, He J, Han P, Tian D (2012). Upregulation of SATB1 promotes tumor growth and metastasis in liver cancer. Liver Int.

[50] Choi EJ, Yun JA, Jabeen S, Jeon EK, Won HS, Ko YH, Kim SY (2014). Prognostic significance of TMEM16A, PPFIA1, and FADD expression in invasive ductal carcinoma of the breast. World J Surg Oncol.

[51] Bandyopadhyay S, Pai SK, Gross SC, Hirota S, Hosobe S, Miura K, Saito K, Commes T, Hayashi S, Watabe M (2003). The Drg-1 gene suppresses tumor metastasis in prostate cancer. Cancer Res.

[52] Bandyopadhyay S, Wang Y, Zhan R, Pai SK, Watabe M, Iiizumi M, Furuta E, Mohinta S, Liu W, Hirota S (2006). The tumor metastasis suppressor gene Drg-1 down-regulates the expression of activating transcription factor 3 in prostate cancer. Cancer Res.

[53] Maruyama Y, Ono M, Kawahara A, Yokoyama T, Basaki Y, Kage M, Aoyagi S, Kinoshita H, Kuwano M (2006). Tumor growth suppression in pancreatic cancer by a putative metastasis suppressor gene Cap43/NDRG1/Drg-1 through modulation of angiogenesis. Cancer Res.

[54] Cheng J, Xie HY, Xu X, Wu J, Wei X, Su R, Zhang W, Lv Z, Zheng S, Zhou L (2011). NDRG1 as a biomarker for metastasis, recurrence and of poor prognosis in hepatocellular carcinoma. Cancer Lett.

[55] Chang JT, Wang HM, Chang KW, Chen WH, Wen MC, Hsu YM, Yung BY, Chen IH, Liao CT, Hsieh LL (2005). Identification of differentially expressed genes in oral squamous cell carcinoma (OSCC): overexpression of NPM, CDK1 and NDRG1 and underexpression of CHES1. Int J Cancer.

[56] Grover PK, Cummins AG, Price TJ, Roberts-Thomson IC, Hardingham JE (2014). Circulating tumour cells: the evolving concept and the inadequacy of their enrichment by EpCAM-based methodology for basic and clinical cancer research. Ann Oncol.

[57] Gastl G, Spizzo G, Obrist P, Dunser M, Mikuz G (2000). Ep-CAM overexpression in breast cancer as a predictor of survival. Lancet.

[58] Ni J, Cozzi P, Hao J, Beretov J, Chang L, Duan W, Shigdar S, Delprado W, Graham P, Bucci J (2013). Epithelial cell adhesion molecule (EpCAM) is associated with prostate cancer metastasis and chemo/radioresistance via the PI3K/Akt/mTOR signaling pathway. Int J Biochem Cell Biol.

[59] Driemel C, Kremling H, Schumacher S, Will D, Wolters J, Lindenlauf N, Mack B, Baldus SA, Hoya V, Pietsch JM (2014). Context-dependent adaption of EpCAM expression in early systemic esophageal cancer. Oncogene.

[60] Romeu C, Farre X, Cardesa A, Nadal A (2013). Expression of ep-CAM, but not of E48, associates with nodal involvement in advanced squamous cell carcinomas of the larynx. Histopathology.

