# Supplementary figures and images for: Prognostic value of biomarkers EpCAM and αB-crystallin associated with lymphatic metastasis in breast cancer by iTRAQ analysis
Source: BMC Cancer. 2019 Aug 23;19:831. doi: 10.1186/s12885-019-6016-3 (PMC6708189; doi:10.1186/s12885-019-6016-3)

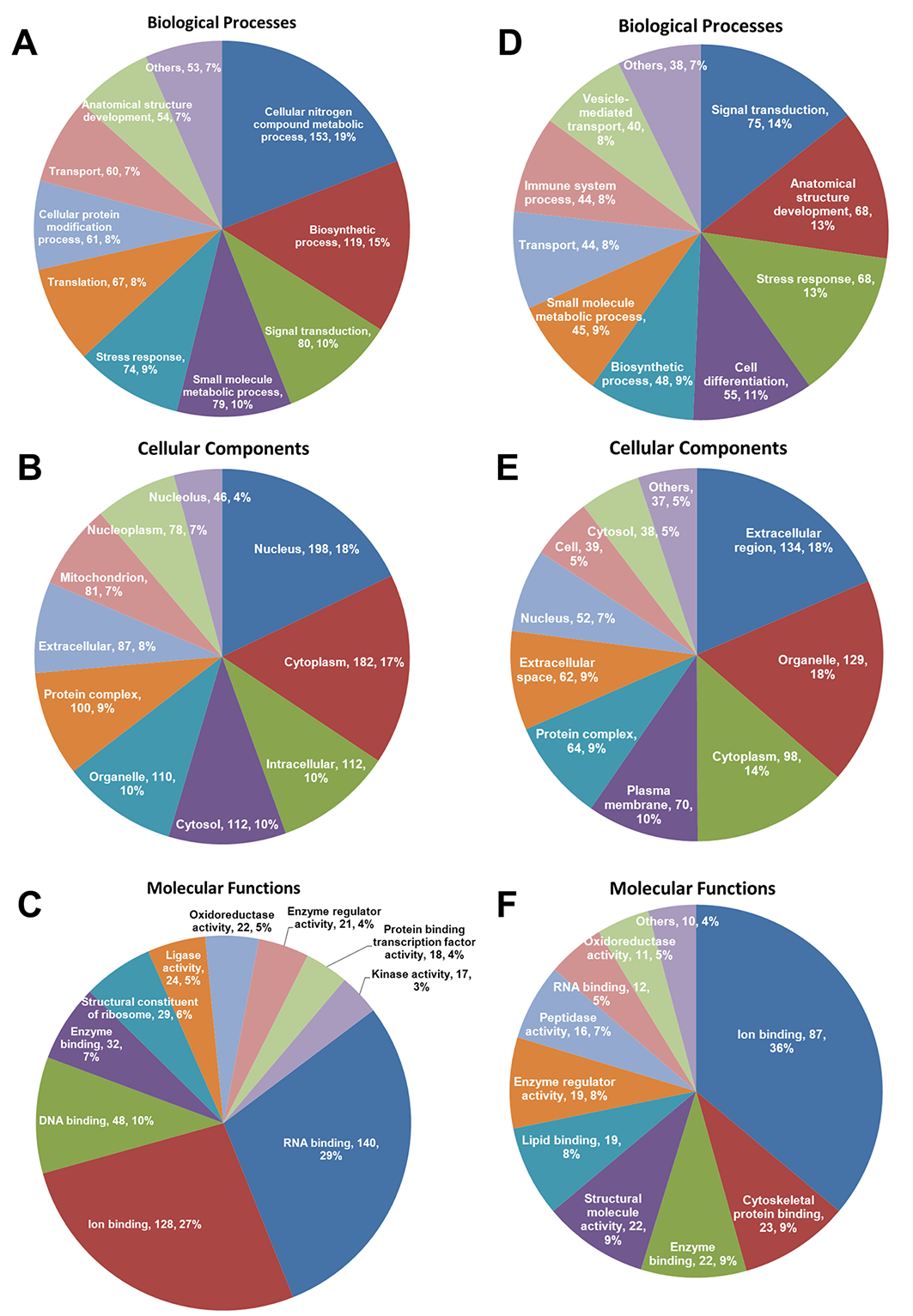

Supplement: Supplementary file 1 — Figure S1. GO analysis of the differentially regulated proteins in lymph node metastases vs. primary breast tumor tissues. The upregulated (A-C) and downregulated (D-F) proteins identified by the iTRAQ proteomics were analyzed by the GO Consortium and categorized according to their biological processes, cellular locations, and molecular functions. (TIF 5559 kb) [file 12885_2019_6016_MOESM1_ESM.tif]

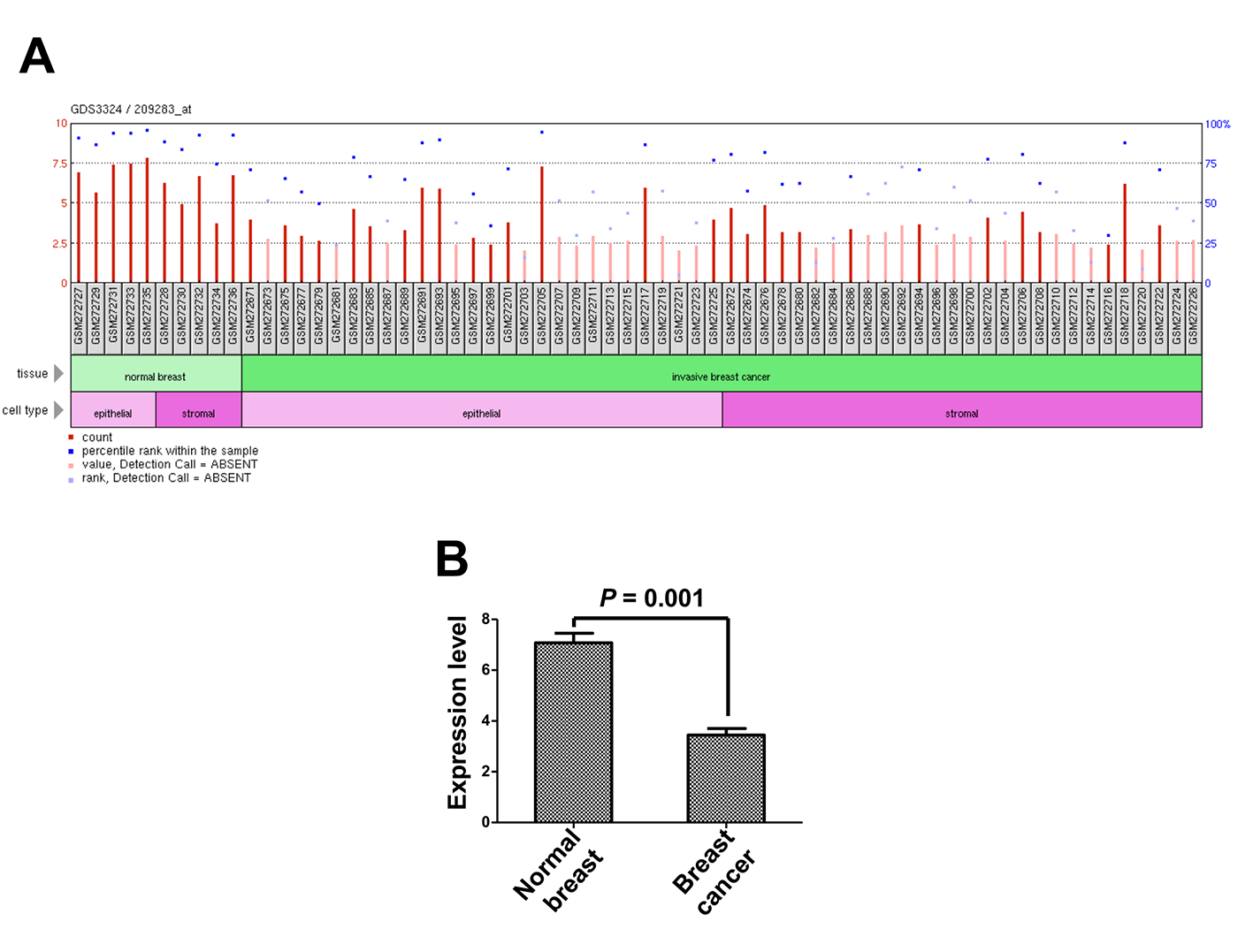

Supplement: Supplementary file 2 — Figure S2. GEO analysis of CRYAB mRNA expression in normal breast and breast cancer tissues. (A) The mRNA expression of CRYAB in normal breast tissues (n =5) and breast cancer tissues (n = 28) was analyzed from the Affymetrix Human Genome Microarray at the GEO website (https://www.ncbi.nlm.nih.gov/geoprofiles/54408377 for αB-crystallin). (B) Quantification of the mRNA expression of CRYAB in normal breast tissues and breast cancer tissues. (TIF 4929 kb) [file 12885_2019_6016_MOESM2_ESM.tif]
