# Supplementary material for: Prognostic value of biomarkers EpCAM and αB-crystallin associated with lymphatic metastasis in breast cancer by iTRAQ analysis
Source: BMC Cancer. 2019 Aug 23;19:831. doi: 10.1186/s12885-019-6016-3 (PMC6708189; doi:10.1186/s12885-019-6016-3)
Supplement: Supplementary file 4 — Table S2. Partial up-regulated proteins in metastatic lymph node compared with primary tumor in breast cancer. Table S3. Partial down-regulated proteins in metastatic lymph node compared with primary tumor in breast cancer. Table S4. UniProt analysis of the biological processes, cellular locations, and molecular functions of the four metastasis-associated proteins. (DOCX 29 kb) [file 12885_2019_6016_MOESM4_ESM.docx]

**Table** S**2.** Partial up-regulated proteins in metastatic lymph node compared with primary tumor in breast cancer.

| Order number | Protein name | Accession  number | Sequence  coverage (%) | Molecular weight | Isoelectric point | Ratio |
| --- | --- | --- | --- | --- | --- | --- |
| 1 | Voltage-dependent anion-selective channel protein 1 | sp\|P21796\|VDAC1_HUMAN | 80.21 | 30773 | 8.79 | 1.87 |
| 2 | Uncharacterized protein | tr\|H7BZT4\|H7BZT4_HUMAN | 65.26 | 10839 | 5.16 | 1.87 |
| 3 | 28S ribosomal protein S34, mitochondrial | tr\|C9JJ19\|C9JJ19_HUMAN | 41.78 | 26332 | 10.4 | 1.87 |
| 4 | Peptidyl-prolyl cis-trans isomerase A | sp\|P62937\|PPIA_HUMAN | 87.88 | 18012 | 7.73 | 1.87 |
| 5 | Apolipoprotein O-like | sp\|Q6UXV4\|APOOL_HUMAN | 26.87 | 29159 | 10.07 | 1.87 |
| 6 | StAR-related lipid transfer protein 7, mitochondrial | sp\|Q9NQZ5\|STAR7_HUMAN | 20.81 | 43113 | 9.23 | 1.86 |
| 7 | Nicotinamide riboside kinase 1 | tr\|Q5W125\|Q5W125_HUMAN | 16.26 | 23797 | 5.14 | 1.86 |
| 8 | Isoform 3 of Protein SEC13 homolog | sp\|P55735-3\|SEC13_HUMAN | 51.63 | 40747 | 5.69 | 1.86 |
| 9 | Splicing factor 3A subunit 3 | sp\|Q12874\|SF3A3_HUMAN | 40.32 | 58849 | 5.05 | 1.86 |
| 10 | ***Epithelial cell adhesion molecule** | sp\|P16422\|EPCAM_HUMAN | 41.72 | 34932 | 7.4 | 1.85 |
| 11 | Isoform 4 of Ubiquitin carboxyl-terminal hydrolase isozyme L5 | sp\|Q9Y5K5-4\|UCHL5_HUMAN | 58.59 | 37353 | 4.98 | 1.84 |
| 12 | Leucine-rich repeat flightless-interacting protein 2 | sp\|Q9Y608\|LRRF2_HUMAN | 33.56 | 82171 | 6.91 | 1.84 |
| 13 | 28S ribosomal protein S18b, mitochondrial | sp\|Q9Y676\|RT18B_HUMAN | 23.64 | 29396 | 9.73 | 1.84 |
| 14 | Protein disulfide-isomerase A6 | tr\|B5MCQ5\|B5MCQ5_HUMAN | 54.29 | 48121 | 4.95 | 1.84 |
| 15 | E3 SUMO-protein ligase RanBP2 | sp\|P49792\|RBP2_HUMAN | 34.34 | 358199 | 6.1 | 1.83 |
| 16 | High mobility group protein B2 | sp\|P26583\|HMGB2_HUMAN | 68.9 | 24034 | 7.78 | 1.83 |
| 17 | Conserved oligomeric Golgi complex subunit 1 (Fragment) | tr\|E9PBL8\|E9PBL8_HUMAN | 23.91 | 107277 | 7.81 | 1.83 |
| 18 | m7GpppX diphosphatase | sp\|Q96C86\|DCPS_HUMAN | 47.18 | 38609 | 6.34 | 1.83 |
| 19 | Aspartate--tRNA ligase, cytoplasmic | sp\|P14868\|SYDC_HUMAN | 65.67 | 57136 | 6.51 | 1.83 |
| 20 | 39S ribosomal protein L24, mitochondrial | sp\|Q96A35\|RM24_HUMAN | 37.04 | 24915 | 9.71 | 1.83 |
| 21 | Thyroid receptor-interacting protein 11 | sp\|Q15643\|TRIPB_HUMAN | 34.51 | 227586 | 4.93 | 1.52 |
| 22 | Interferon-inducible double-stranded RNA-dependent protein kinase activator A | sp\|O75569\|PRKRA_HUMAN | 37.06 | 34404 | 8.48 | 1.52 |
| 23 | Biorientation of chromosomes in cell division protein 1-like | sp\|Q8NFC6\|BD1L1_HUMAN | 16.58 | 330466 | 4.73 | 1.52 |
| 24 | Cleavage stimulation factor subunit 2 | sp\|P33240\|CSTF2_HUMAN | 33.79 | 60959 | 6.78 | 1.52 |
| 25 | Proteasome subunit beta type-2 | sp\|P49721\|PSB2_HUMAN | 64.68 | 22836 | 6.96 | 1.52 |
| 26 | ***FAS-associated death domain protein** | sp\|Q13158\|FADD_HUMAN | 45.19 | 23279 | 5.39 | 1.51 |
| 27 | Transmembrane emp24 domain-containing protein 2 | sp\|Q15363\|TMED2_HUMAN | 45.27 | 22761 | 4.83 | 1.51 |
| 28 | Estradiol 17-beta-dehydrogenase 12 | sp\|Q53GQ0\|DHB12_HUMAN | 47.44 | 34324 | 9.79 | 1.51 |
| 29 | Ubiquinone biosynthesis protein COQ7 homolog | sp\|Q99807\|COQ7_HUMAN | 33.18 | 24277 | 8.67 | 1.51 |
| 30 | Proliferation-associated protein 2G4 | sp\|Q9UQ80\|PA2G4_HUMAN | 59.64 | 43787 | 6.51 | 1.51 |

**Table** S3**.** Partial down-regulated proteins in metastatic lymph node compared with primary tumor in breast cancer.

| Order number | Protein name | Accession number | Sequence coverage (%) | Molecular weight | Isoelectric point | Ratio |
| --- | --- | --- | --- | --- | --- | --- |
| 1 | Isoform 4 of Gelsolin | sp\|P06396-4\|GELS_HUMAN | 69.81 | 81485 | 5.6 | 0.27 |
| 2 | Complement C5 | sp\|P01031\|CO5_HUMAN | 27.73 | 188305 | 6.49 | 0.27 |
| 3 | Kallistatin | sp\|P29622\|KAIN_HUMAN | 50.81 | 48542 | 7.71 | 0.28 |
| 4 | Ig lambda-2 chain C regions | sp\|P0CG05\|LAC2_HUMAN | 89.62 | 11294 | 7.1 | 0.28 |
| 5 | Complement factor H-related protein 1 | sp\|Q03591\|FHR1_HUMAN | 29.39 | 37651 | 7.35 | 0.28 |
| 6 | Olfactomedin-like protein 3 | sp\|Q9NRN5\|OLFL3_HUMAN | 41.87 | 46010 | 6.51 | 0.29 |
| 7 | Transthyretin | sp\|P02766\|TTHY_HUMAN | 68.7 | 15887 | 5.57 | 0.29 |
| 8 | Uncharacterized protein (Fragment) | tr\|S4R394\|S4R394_HUMAN | 43.47 | 12477 | 8.25 | 0.3 |
| 9 | Isoform 2 of 72 kDa type IV collagenase | sp\|P08253-2\|MMP2_HUMAN | 20.89 | 65765 | 4.83 | 0.31 |
| 10 | Long-chain-fatty-acid--CoA ligase 1 | tr\|E7EPM6\|E7EPM6_HUMAN | 38.1 | 74282 | 7.29 | 0.31 |
| 11 | Ig lambda chain V-III region LOI | sp\|P80748\|LV302_HUMAN | 37.83 | 11935 | 4.72 | 0.31 |
| 12 | Isoform 2 of Hormone-sensitive lipase | sp\|Q05469-2\|LIPS_HUMAN | 24.39 | 84128 | 6.68 | 0.32 |
| 13 | Protein kinase C delta-binding protein | tr\|E9PIE3\|E9PIE3_HUMAN | 40.27 | 31088 | 6.77 | 0.32 |
| 14 | Tropomyosin alpha-4 chain | sp\|P67936\|TPM4_HUMAN | 83.46 | 28522 | 4.36 | 0.32 |
| 15 | SH3 and PX domain-containing protein 2B | sp\|A1X283\|SPD2B_HUMAN | 24.36 | 101579 | 9.04 | 0.33 |
| 16 | Adipocyte enhancer-binding protein 1 | sp\|Q8IUX7\|AEBP1_HUMAN | 39.37 | 130929 | 4.77 | 0.33 |
| 17 | Complement factor I light chain | tr\|G3XAM2\|G3XAM2_HUMAN | 29.85 | 65060 | 7.49 | 0.33 |
| 18 | ***Protein NDRG1** | sp\|Q92597\|NDRG1_HUMAN | 45.17 | 42835 | 5.67 | 0.33 |
| 19 | Insulin-like growth factor-binding protein complex acid labile subunit | sp\|P35858\|ALS_HUMAN | 19.16 | 66035 | 6.76 | 0.33 |
| 20 | ***Alpha-crystallin B chain (Fragment)** | tr\|E9PR44\|E9PR44_HUMAN | 53.45 | 20031 | 6.99 | 0.34 |
| 21 | Ig alpha-2 chain C region | sp\|P01877\|IGHA2_HUMAN | 37.34 | 36526 | 6.02 | 0.34 |
| 22 | Ig heavy chain V-III region GAL | sp\|P01781\|HV320_HUMAN | 70.69 | 12730 | 4.57 | 0.34 |
| 23 | Isoform 1 of Vinculin | sp\|P18206-2\|VINC_HUMAN | 71.57 | 116722 | 5.87 | 0.35 |
| 24 | Complement C1q subcomponent subunit B | tr\|D6R934\|D6R934_HUMAN | 50.19 | 26459 | 8.77 | 0.35 |
| 25 | Isoform 2 of Peptidyl-prolyl cis-trans isomerase FKBP7 | sp\|Q9Y680-2\|FKBP7_HUMAN | 42.34 | 25794 | 6.25 | 0.35 |
| 26 | Ig gamma-3 chain C region | sp\|P01860\|IGHG3_HUMAN | 91.25 | 41287 | 7.89 | 0.36 |
| 27 | Aminopeptidase N | sp\|P15144\|AMPN_HUMAN | 21.19 | 109540 | 5.14 | 0.36 |
| 28 | Arf-GAP domain and FG repeat-containing protein 2 | sp\|O95081\|AGFG2_HUMAN | 10.18 | 48963 | 9.5 | 0.37 |
| 29 | Retinol-binding protein 4 | sp\|P02753\|RET4_HUMAN | 51.23 | 23010 | 5.84 | 0.37 |
| 30 | Ig kappa chain V-I region Mev | sp\|P01612\|KV120_HUMAN | 53.21 | 11870 | 6.17 | 0.37 |

**Table S4.** UniProt analysis of the biological processes, cellular locations, and molecular functions of the four metastasis-associated proteins.
